# Epidemiology, clinical features and risk factors for human rabies and animal bites during an outbreak of rabies in Maputo and Matola cities, Mozambique, 2014: Implications for public health interventions for rabies control

**DOI:** 10.1371/journal.pntd.0005787

**Published:** 2017-07-24

**Authors:** Cristolde Salomão, Amílcar Nacima, Lutero Cuamba, Lorna Gujral, Olga Amiel, Cynthia Baltazar, Julie Cliff, Eduardo Samo Gudo

**Affiliations:** 1 Department of Surveillance, National Institute of Health, Maputo, Mozambique; 2 Field Epidemiology and Laboratory Training Program, National Institute of Health, Maputo, Mozambique; 3 Maputo City Health Directorate, Ministry of Health, Maputo, Mozambique; 4 Department of Epidemiology, National Directorate of Public Health, Ministry of Health, Maputo, Mozambique; 5 Department of Neglected Tropical Diseases Program, National Directorate of Public Health, Maputo, Mozambique; 6 Faculty of Medicine, Eduardo Mondlane University, Maputo, Mozambique; Universidad Nacional Mayor de San Marcos, PERU

## Abstract

**Background:**

In Mozambique, the majority of rabies outbreaks are unreported and data on the epidemiological features of human rabies and animal bites are scarce. An outbreak of human rabies in adjacent Maputo and Matola cities in 2014 prompted us to investigate the epidemiology, clinical features and risk factors of human rabies and animal bites in the two cities.

**Methodology/Principal findings:**

We reviewed cases of human rabies and animal bites from April to July 2014, and carried out a community investigation in July and August in the neighborhoods where cases of human rabies resided. This investigation included collection of clinical, demographic and epidemiological information and a case control study to investigate the risk factors associated with human rabies. Fourteen cases of human rabies were detected in Maputo (n = 10) and Matola (n = 3) cities and neighbouring Boane district (n = 1) between April and August 2014, all of whom had been admitted to hospital. All had a recent history of dog bite. Of the 14 rabid dogs, only one had been immunized. 819 cases of animal bites were registered, of which 64.6% (529/819) were from Maputo City. Dogs were responsible for 97.8% (801/819) of all animal bites, but only 27.0% (126/467) were immunized. Factors significantly associated with human rabies were: age <15 years (p = 0.05), bite by stray dog (p = 0.002), deep wound (p = 0.02), bite in the head (p = 0.001), bite by unimmunized dog (p = 0.01), no use of soap and water (p = 0.001), and no post-exposure prophylaxis (p = 0.01).

**Conclusions/Significance:**

Implementation of control measures for rabies is poor in Maputo and Matola cities, where cases of human rabies were strongly associated with bites by stray and unvaccinated dogs and irregular implementation of post-exposure measures.

## Author summary

Rabies is a deadly neglected virus that causes an estimated 59,000 deaths each year worldwide. In 2014, an outbreak of human rabies in adjacent Maputo and Matola cities prompted us to investigate the epidemiology and risk factors of human rabies and animal bites in the two cities. We reviewed cases of human rabies and animal bites through both health facility and community investigations and carried out a case control study in affected neighborhoods. We found a total of 14 unreported human rabies cases in a four month period, and 819 cases of animal bites in a three month retrospective review of records. Dogs were responsible for all rabid bites and for 97.8% of animal bites. None of the rabid dogs had been vaccinated. Factors significantly associated with human rabies were: age <15 years, bite by a stray dog, deep wound, bite in the head, bite by an unimmunized dog, failure to use soap and water to cleanse the wound, and to vaccinate after the bite. We concluded that the poor implementation of control measures for rabies had led to the outbreak and recommended strengthening implementation, particularly in rapidly urbanizing areas.

## Introduction

Rabies is a zoonotic viral disease, belonging to the *Lyssavirus* genus of the *Rhabdoviridae* family, which infects mammals and causes fatal encephalitis [1, 2]. A bite by a rabid dog represents the source of infection in more than 99% of cases [3, 4]. Almost half of cases occur in children under the age of 15 years [3, 5, 6]. In humans, the incubation period of rabies is highly variable, ranging from days to years, with an average of 2–3 months, being influenced by: i) location, extent and depth of the wound; ii) distance between the location of the wound and the central nervous system; iii) concentration of inoculated virus particles and iv) virus strain [1, 6, 7]. The incubation period in children tends to be of shorter duration than adults [7].

Although rabies is preventable, and effective control measures are available, the number of deaths is still high and an estimated 59,000 people die from rabies each year in the world, with Asia and sub-Saharan Africa accounting for more than 95% of these deaths [8]. More than 3.3 billion people are at risk of infection by the rabies virus [3].

Rabies disproportionally affects the poorest and under-resourced populations living in low and middle income countries, and its incidence is on the rise in several countries (8–15). In addition to its negative impact on mortality, rabies also causes a negative economic impact in already impoverished populations (8, 16). Lack of reliable data on the burden and risk factors associated with human rabies represents a critical challenge for the formulation of policies and strategies to control the disease and has been considered a major cause for the under investment in rabies control measures in these countries (8, 20).

With regard to Mozambique, recent estimates of the burden of rabies in sub-Saharan Africa ranked Mozambique as one of the countries with the highest burden [8]. In Mozambique, rabies remains badly neglected.

Mozambique elaborated a national rabies control strategy in 2010 (9). Control activities and responsibilities are shared by the Ministry of Agriculture, Ministry of Health, Municipalities (city governments) and local authorities. The main interventions are: i) immunization of domestic pets, mostly dogs; ii) removal and euthanizing of all stray and unowned dogs and iii) timely and appropriate implementation of post-exposure prophylaxis, including washing of the bite site and post-exposure vaccination (1, 6).

Veterinary immunization is mostly provided by the Public Veterinary Services in coordination with the local municipalities; private veterinary services also provide vaccination, but on a limited scale. The municipalities are also responsible for the removal of stray dogs (21). Post-exposure vaccination and rabies immunoglobulin (RIG) are offered at specialized prevention facilities, named Prophylaxis Centers, which are scattered throughout the country (21). All victims of animal bites are referred to these centers for five doses of prophylactic inactivated rabies vaccine produced in cell culture, and RIG, as per the WHO protocol. Conduct regarding the animal follows national and international procedures. Owners are advised to quarantine all biting animals in the household for ten days and to return to the center if the animal develops any sign of rabies (1, 6). At ten days they must return to the center for a physical examination of the animal by a health professional (1, 21, 36). However, in Mozambique, as in many sub-Saharan Africa countries, these procedures are conducted irregularly, resulting in frequent cases or outbreaks of rabies.

In Maputo City, the national capital, health authorities were alerted to a possible outbreak of rabies in July 2014 when Maputo Central Hospital admitted several patients with rabies in a short time period. This outbreak prompted further investigation of both rabies cases and animal bites in the city and adjoining Matola City, capital of Maputo Province. To our knowledge this represents the first ever attempt to investigate a rabies outbreak and determine the epidemiological profile of animal bites in Mozambique.

We aimed to determine the clinical and epidemiological features of human rabies cases detected during the outbreak, the risk factors for developing rabies, and the epidemiological features of animal bites in Maputo and Matola cities We expect that the findings of this study will contribute to formulating appropriate recommendations and strategies to control rabies in Mozambique.

## Materials and methods

### Ethics statement

A rabies outbreak is a public health emergency and this investigation was part of the public health response. For this reason, no protocol was submitted to local IRB and the investigation was exempt from ethical approval, but permission to conduct the investigation was obtained from local health authorities and local community leaders. In addition, the investigation team ensured that a comprehensive explanation of the investigation was provided to all health professionals and other participants. A verbal consent was obtained from each participant or their parents and/or legal guardians before the interview and also to use their questionnaire forms.

### Study setting

**Fig 1:**
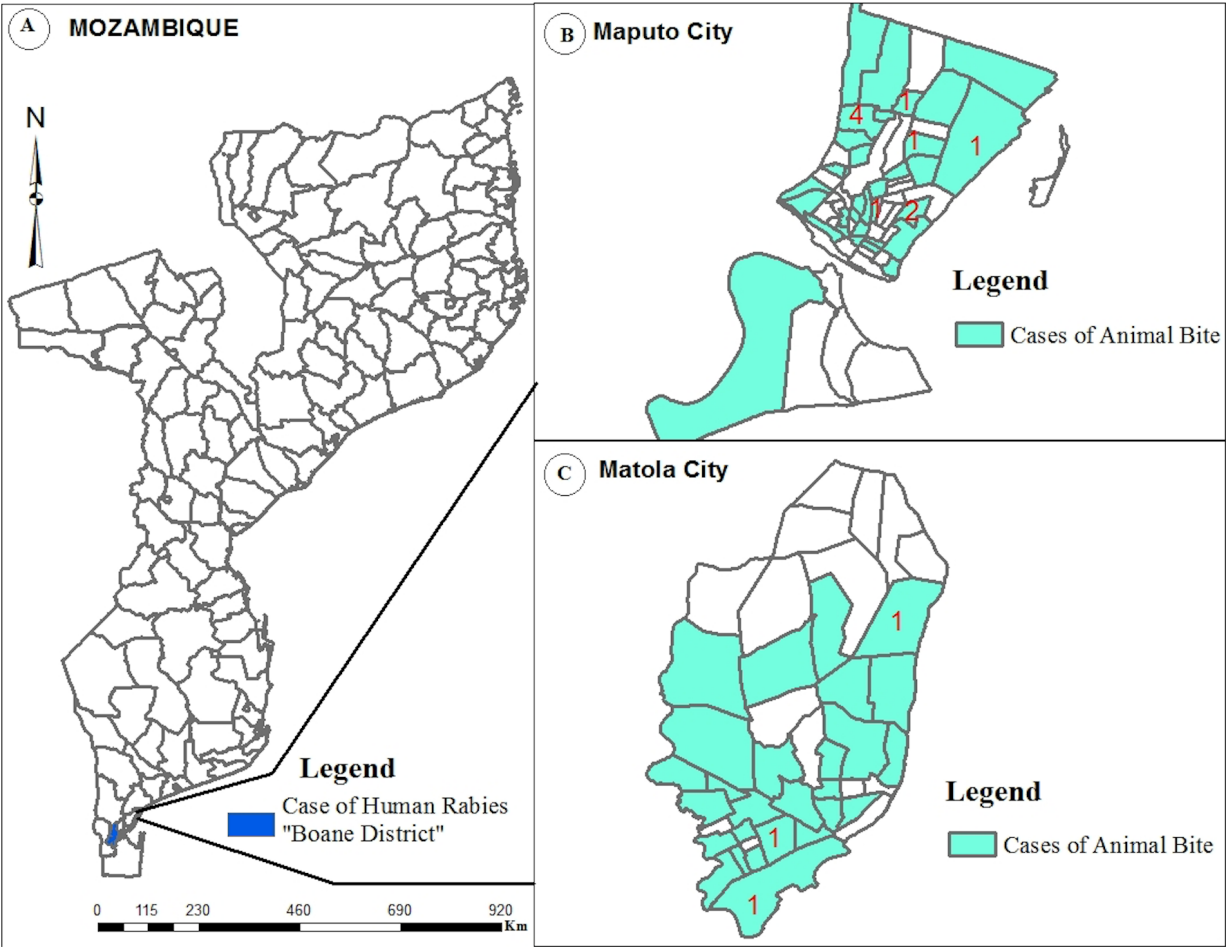
Geographical distribution of cases of animal bites and human rabies in Maputo and Matola cities between April and July 2014. Panel A depicts the Mozambique map and the study place is highlighted in blue color. Panel B shows Maputo city in higher magnification. Panel C shows Maputo province in higher magnification. In panel B and C, red numbers represent neighborhoods where cases of human rabies occurred and green color highlight neighborhood where dog bites were reported.

Maputo City is the capital of Mozambique, with an area of 347 km^2^ and a population estimated at 1,225,868 inhabitants [9]. The city is divided into seven districts, which are in turn divided into 61 neighborhoods. An estimated 70% of inhabitants live in the suburbs, often in overcrowded housing with poor sanitation and a low education level. A small proportion live in the central urban (13.8%) and peri-urban (8.7%) areas [9]. Matola City, the capital of Maputo Province, adjoins Maputo City, and has an area of 373 km^2^ and a population estimated at 893,000 inhabitants [9]. Matola City is divided into 3 administrative posts and 42 neighborhoods. Although the vast majority of the population lives in peri-urban or semi-rural areas, the city has a rapid growth rate and is rapidly urbanizing. Maputo and Matola cities have several Prophylaxis Centers, to which all animal bites are referred. Both cities are situated in the southern part of the country (Fig 1). Notifications of rabies cases in Maputo City increased from one in 2013 to 21 in 2014 (22). The incidence of notified cases of dog bites per inhabitant in Maputo City is the highest in the country (17).

### Control measures

Immediately after the notification of the outbreak, the following control measures were instituted: i) establishment of a coordination committee comprising the National Veterinary Services in the Ministry of Agriculture, National Institute of Health and the National Directorate for Public Health in the Ministry of Health, Maputo City Council, Maputo Province government and local communities; ii) affixing posters in health units regarding treatment of animal bites and post- exposure prophylaxis; iii) delivery of additional quantities of anti-rabies vaccine to the Prophylaxis Centers in Maputo City and Province to ensure an adequate stock; iv) decentralization of post-exposure prophylaxis to Matola 2 and Boane health centers; v) vaccination of dogs in the neighborhoods where human rabies cases had occurred; vi) mass vaccination campaign of dogs in Maputo City and Maputo Province; vii) Participation of private veterinary clinics in animal vaccination; viii) collection of stray dogs in selected neighborhoods; ix) community education regarding prevention and control measures.

### The investigation

#### Detection and characterization of human rabies cases in Maputo Central Hospital

The study was initiated as part of the epidemiological investigation of a suspected rabies outbreak, immediately after Ministry of Health officials were alerted by clinicians at Maputo Central Hospital that three children had been admitted between June 30^th^ and July 9^th^, 2014, with suspected rabies following recent dog bites. All of the children died shortly after admission. We immediately collected clinical and epidemiological details and information on post-exposure prophylaxis on each deceased child from the clinical files, and also interviewed attending physicians and parents. Maputo Central Hospital is a quaternary hospital situated in Maputo City and is the referral hospital for the whole country. Most cases of rabies from Maputo and Matola Cities are referred to this hospital. Three further cases admitted in July were included in the investigation.

We retrospectively searched for additional cases of human rabies admitted to Maputo Central Hospital from the 1^st^ April 2014 in the admission logbooks of the Pediatric and Adult Medical Wards and the autopsy logbook of the Forensic Services.

We collected clinical and epidemiological details, and information on possible risk factors in each case from the clinical records. When available, autopsy records were also examined.

#### Case definition and diagnosis of human rabies

The diagnosis of rabies was performed by experienced pediatricians and an experienced internal medicine specialist, for children and adults, respectively. Diagnosis was based on a combination of clinical findings and epidemiological history, without laboratory confirmation. Rabies was defined as the presence of clinical symptoms and signs suggestive of rabies, and a rapid progression to death associated with a recent history of animal bite.

#### Community survey of rabies cases and animal bites

A community investigation was conducted between July and August with the aim of identifying all cases of animal bites, additional cases of human rabies and additional victims who had been exposed to the rabid animal. The investigation was conducted in the suburban areas of Maputo and Matola cities, as all except one of the fatal bites resided in these suburbs (all fatal cases were bitten in the neighborhood where the victim resided). Although one of the victims who developed rabies resided in Boane District, this district was not included in the community investigation. We included not only the neighborhoods where fatal cases resided, but also the surrounding neighborhoods. Local leaders were contacted and participated in the investigation, providing information on households to be interviewed. Cases of bites identified in the community were referred for post-exposure prophylaxis.

We interviewed the parents or the legal representative of the rabies and animal bite victims using a standard questionnaire, which included clinical, demographic and risk factors.

The following information was collected: age and gender of victim, residence, date, location and severity of bite, type and ownership of animal, depth of the wound, site of bite, immunization status of the biting animal, cleaning of the wound after the bite and post-exposure vaccination of the victims.

#### Case control study

A case control study was conducted to investigate the risk factors associated with human rabies. For each case of human rabies, 4 cases of animal bites situated in the same neighborhood close to the human rabies victim’s house were used as controls (case: control ratio of 1:4). Age and gender were not eligibility criteria for the control group. The same standard questionnaire on demographic variables and other known risk factors was used (as detailed above).

#### Laboratory diagnosis of rabies in animals

All suspected animals were sent to the Central Veterinary Laboratory in Maputo City for laboratory confirmation using a fluorescent antibody test. Sections of autopsied brain samples were stained using anti-rabies serum or globulin labeled with fluorescein isothiocyanate (FITC) and the presence of rabies virus nucleoprotein antigen was visualized using fluorescent microscopy [10].

#### Retrospective analysis of animal bites at Prophylaxis Centers

We collected details of all cases of animal bites recorded at the Prophylaxis Centers in Maputo and Matola Cities between April and July 2014. At the Prophylaxis Centers, each case of animal bite is recorded in a logbook and a case investigation form is filled out. Both documents were used as a source of data.

#### Comparison with routine surveillance

Both animal bites and rabies deaths are notified weekly through the routine surveillance system, which covers all public health facilities in the country. We compared the numbers of animal bites and rabies cases found in our investigation from April to July 2014 with those notified by routine surveillance.

### Statistical analysis

All forms were anonymized. Data were analyzed using the statistics package STATA 12.1 (College Station, Texas: Stata Corp, USA, 2012). Frequencies, proportions, odds ratio (OR) and chi-square test were calculated for categorical variables. Median, interquartile range (IQR) and Mann-Whitney test were calculated for numerical variables. A p-value <0.05 was considered statistically significant.

Logistic regression analysis was used to determine the variables independently associated with human rabies, controlling for confounders. The analysis was built using backwards stepwise method and Log Likelihood Ratio Test. For this purpose, all variables with a p- value less than 0.25 on univariate analysis were included in the initial multivariate model. A p-value < 0.05 was considered of statistical significance in the final model.

## Results

### Frequency and characteristics of cases of human rabies

We detected a total of 12 cases of fatal rabies admitted to Maputo Central Hospital during the period between April and July 2014. These were comprised of the initial three cases, a further three cases admitted later, and a further six cases identified by retrospective analysis of the medical records. During the community investigation, an additional two cases admitted in August to a different hospital in Maputo were identified. Rabies was not suspected and for this reason, they were not initially recorded as rabies. We have included them in the analysis.

**Fig 2:**
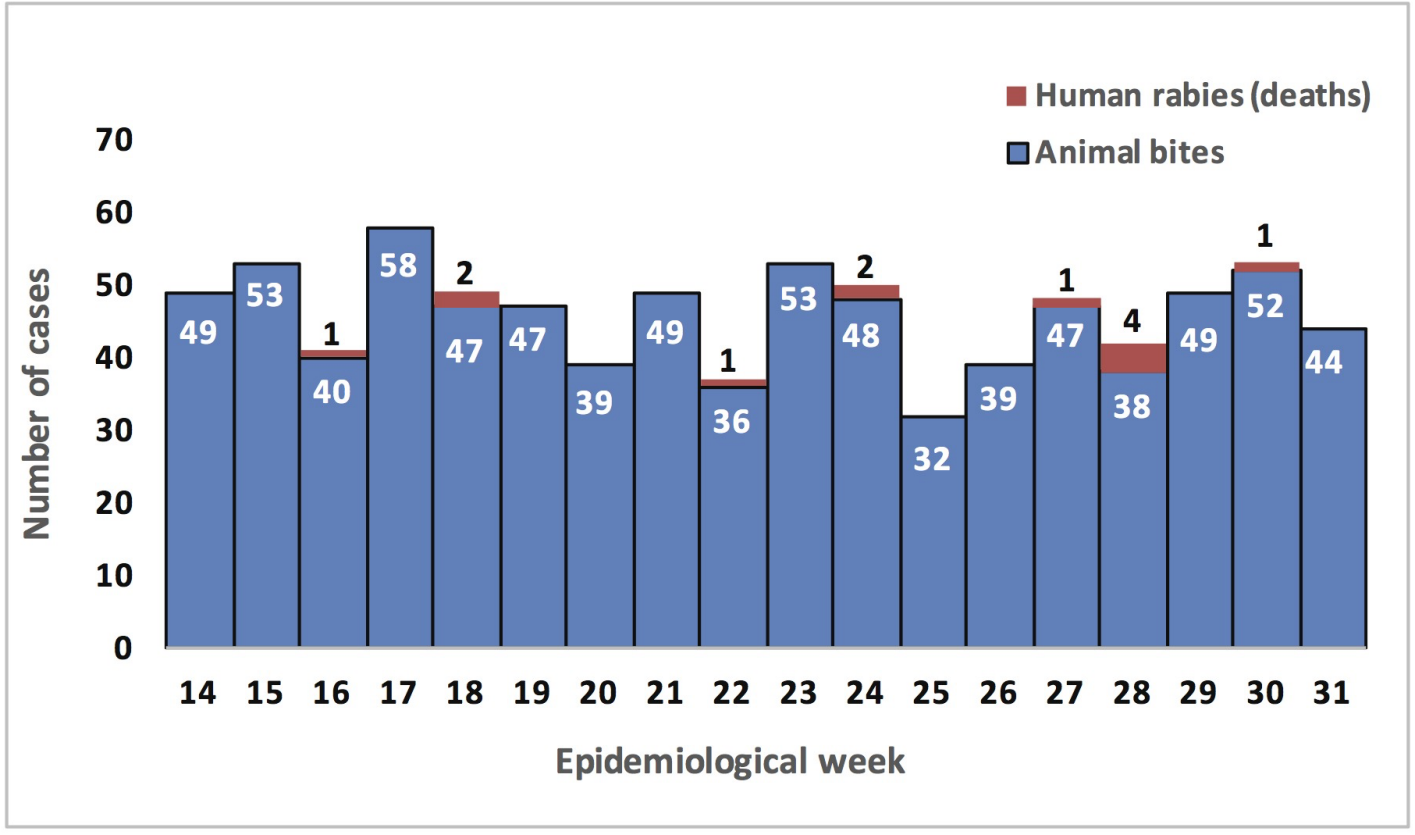
Frequency of cases of animal bite and rabies deaths in Maputo city and Province, April to July 2014.

Table 1 shows that, of the 14 cases of human rabies, 12 were children below 15 years of age and 8 were male. Ten lived in the suburban areas of Maputo City, 3 in Matola City and 1 in Boane District, a rural district in Maputo Province (Fig 1). Half of the deaths occurred between 11^th^ June and 13^th^ July (epidemiological weeks 24 to 28), as shown in Fig 2.

**Table 1:** Clinical and demographic characteristics of human rabies cases during the outbreak between April and August 2014.

Case #	Age (years)	Gender	Residence (neighborhood)	Date of animal bite	Date of admission	# Days between admission and death
1	4	M	Maxaquene	No information	14.04.14	4
2	14	F	Magoanine	25.03.14	28.04.14	2
3	8	M	Polana Caniço B	No information	03.05.14	5
4	29	F	Polana Caniço B	15.02.14	29.05.14	2
5	20	M	Fomento (Matola)	07.04.14	10.06.14	1
6	5	F	George Dimitrov	05.06.14	13.06.14	2
7	6	M	George Dimitrov	04.06.14	30.06.14	1
8	3	F	Laulane	05.06.14	08.07.14	3
9	6	F	Boane District	13.05.13	09.07.14	1
10	10	M	Matola A(Matola)	01.06.14	10.07.14	1
11	7	F	Intaka (Matola)	28.05.14	12.07.14	1
12	2	M	Triunfo	05.06.14	24.07.14	0
13*	8	M	George Dimitrov	05.06.14	04.08.14	0
14*	12	M	George Dimitrov	05.06.14	14.08.14	2

*These two cases are connected epidemiologically with the 6th case, and were identified by active surveillance in the community.

All of the 14 cases of human rabies involved dog bite. Only one of the dogs had been immunized.

The average duration of hospitalization before death was two days, ranging between less than 24 hours up to 5 days, as shown in Table 1.

**Fig 3:**
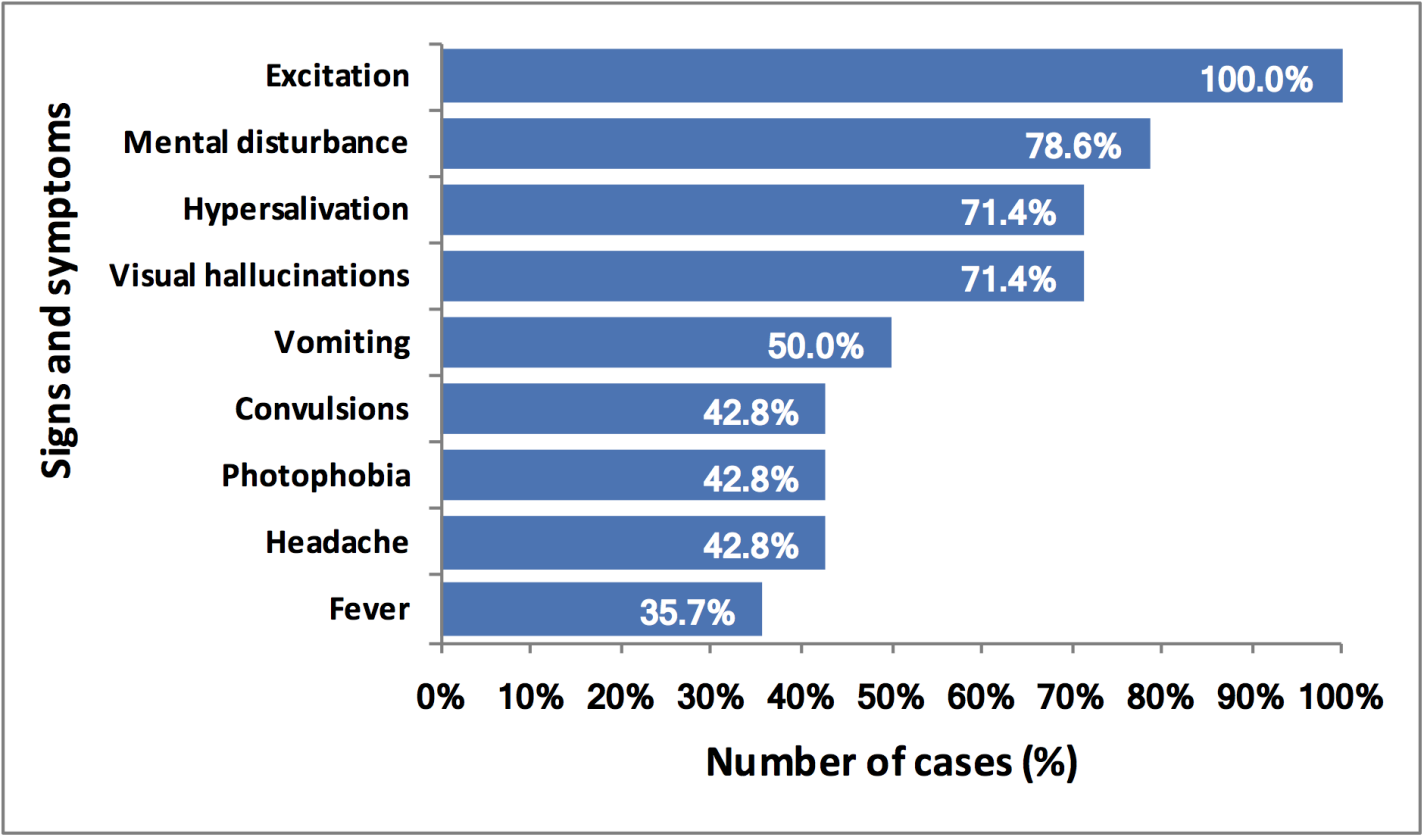
Frequency of signs and symptoms in cases of human rabies.

Main clinical findings before death were excited behavior (100%), mental disturbance (78.6%), visual hallucinations (71.4%) and hypersalivation (71.4%), as shown in Fig 3.

### Post exposure Prophylaxis in rabies cases

No rabies victim received full post-exposure vaccination. In Matola, one victim attended a health center and received only tetanus vaccine and the parents were informed that it was sufficient. Three victims attended the Prophylaxis Center in Matola and the parents were told that there was no vaccine available. In Maputo City, of ten victims who attended health centers, nine were given only tetanus vaccine and parents were told that it was sufficient. They were not referred to the Prophylaxis Center. The one case who was referred was only given two doses of vaccine.

### Frequency and characteristics of cases of animal bites

A total of 819 cases of animal bites were registered during the period under investigation (April to July 2014), 529 (64.6%) in Maputo City and 290 (35.4%) in Matola City, as shown in Table 2. Fig 1 shows that cases came from many different neighborhoods. Fig 2 shows that the number of bites ranged from 32 to 58 cases per week.

**Table 2:** Characteristics of animal bites registered in Maputo and Matola cities, April to July 2014.

Characteristics	Maputo City	Matola City	Total
	n (%)	n (%)	n (%)
**Gender of the victim**	***n = 529***	***n = 290***	***n = 819***
M	314 (59.4)	155 (53.4)	479 (57.3)
F	215 (40.6)	135 (46.6)	350 (42.7)
**Age of the victim, years**			
Median	20	21	20
IQR	10–33	10–35	10–34
Range	1–85	1–86	1–86
**Age group of the victim, years**	***n = 529***	***n = 290***	***n = 819***
0–4	31 (5.8)	16 (5.5)	47 (5.7)
5–14	175 (33.0)	88 (30.3)	263 (32.1)
15–45	252 (47.7)	147 (50.8)	399 (48.7)
46–86	63 (11.9)	34 (11.7)	97 (11.8)
No information	8 (1.5)	5 (1.7)	13 (1.7)
**Residence of the victim**	***n = 529***	***n = 290***	***n = 819***
Central urban	32 (6.0)	90 (31.0)	122 (14.9)
Suburban	497 (94.0)	200 (69.0)	697 (85.1)
**Type of biting animal**	***n = 529***	***n = 290***	***n = 819***
Dog	518 (97.9)	283 (97.6)	801 (97.8)
Cat	5 (0.9)	4 (1.4)	9 (1.1)
Human	5 (0.9)	3 (1.0)	8 (1.0)
Mouse	1 (0.3)	0 (0.0)	1 (0.1)
**Availability of animal bite investigation form**	***n = 529***	***n = 290***	***n = 819***
Yes	40 (7.6)	31 (10.7)	71 (8.7)
No	489 (92.4)	259 (89.3)	748 (91.3)
**Post-exposure prophylaxis of the victim**	***n = 529***	***n = 290***	***n = 819***
Vaccinated	1 (0.2)	46 (15.9)	47 (5.7)
Not vaccinated	11 (2.1)	98 (33.8)	109 (13.3)
No information	517 (97.7)	146 (50.3)	663 (81.0)
**Ownership of the biting dog**	***n = 529***	***n = 290***	***n = 819***
Known or Owned	324 (61.2)	143 (49.3)	467 (57.0)
Stray	88 (16.6)	106 (36.6)	194 (23.7)
Unknown	106 (20.0)	34 (11.7)	140 (17.1)
No information	11 (2.1)	7 (2.4)	18 (2.1)
**Condition of the biting dog 10 days post aggression**	***n = 324***	***n = 143***	***n = 467***
Alive	16 (4.9)	4 (2.8)	20 (4.3)
Natural Death	3 (0.9)	2 (1.4)	5 (1.1)
Killed	2 (0.6)	1 (0.7)	3 (0.6)
Escaped after aggression	3 (0.9)	3 (2.1)	6 (1.3)
No information	300 (92.6)	133 (92.4)	433 (92.7)
**Immunization status of the biting dog**	***n = 324***	***n = 143***	***n = 467***
Immunized	61 (18.8)	65 (45.4)	126 (27.0)
Not immunized	70 (21.6)	38 (26.6)	108 (23.0)
Unknown	193 (59.6)	40 (28.0)	233 (50.0)
**Status of anti-rabies immunization of the biting animal**	***n = 61***	***n = 65***	***n = 126***
Up-to-date or valid	56 (91.8)	60 (92.3)	116 (92.1)
Expired	5 (8.2)	5 (7.7)	10 (7.9)

For each variable, the percentages of each category excluded the missing category.

Table 2 also shows that 57.3% (469/819) of bite victims were male. The median age of victims was 20 years (IQR: 10–34 years), ranging between 1 and 86 years. Children under 15 years of age comprised 37.9% (310/819) of the victims.

In both cities, the majority of animal bites occurred in people living in the suburbs (85.1%; 697/819), with only 14.9% (122/819) living in the central urban area.Most animal bites were caused by dogs (97.8%, 801/819). Small proportions were attributed to cats (1.1%, 9/819), humans (1.0%, 8/819) and mice (0.1%, 1/819).

Only 72 (8.7%) of animal bite victims had an animal bite investigation form filled out and the majority of these forms were incomplete. Most of the information was, therefore, retrieved from the logbooks. Information on post-exposure vaccination was available in only 19.0% (156/819) of victims, of whom 7.7% (12/156) were from Maputo City and 92.3% (144/154) from Matola City. Of the 156 victims for whom information on post-exposure vaccination was available, only 47 (30.1%) received the vaccine, of whom all except one lived in Matola City.

The biting dog was known or owned in 58.3% (467/801) of dog bite cases. In 23.7% (194/801) and 17.1% (140/801) the biting dog was stray or unknown, respectively.

Of the 467 dogs that were owned, only 7.3% (34/467) of the owners provided any information on the clinical condition of the animal 10 days after the bite. Of the total known or owned dogs, 4.3% (20/467) were still alive, 1.1% (5/467) had died a natural death and 0.6% (3/467) were killed.

Of the 467 dog that were known or owned, 27.0% (126/467) were immunized, 23.0% (108/467) were not immunized and for the remaining 50% (233/467) their immunization status was unknown. Among those that were immunized, 92.1% (116/126) had a valid immunization status and in 7.9% (10/126) vaccine validity had expired.

### Epidemiological linkage between animal bite and human rabies

**Fig 4:**
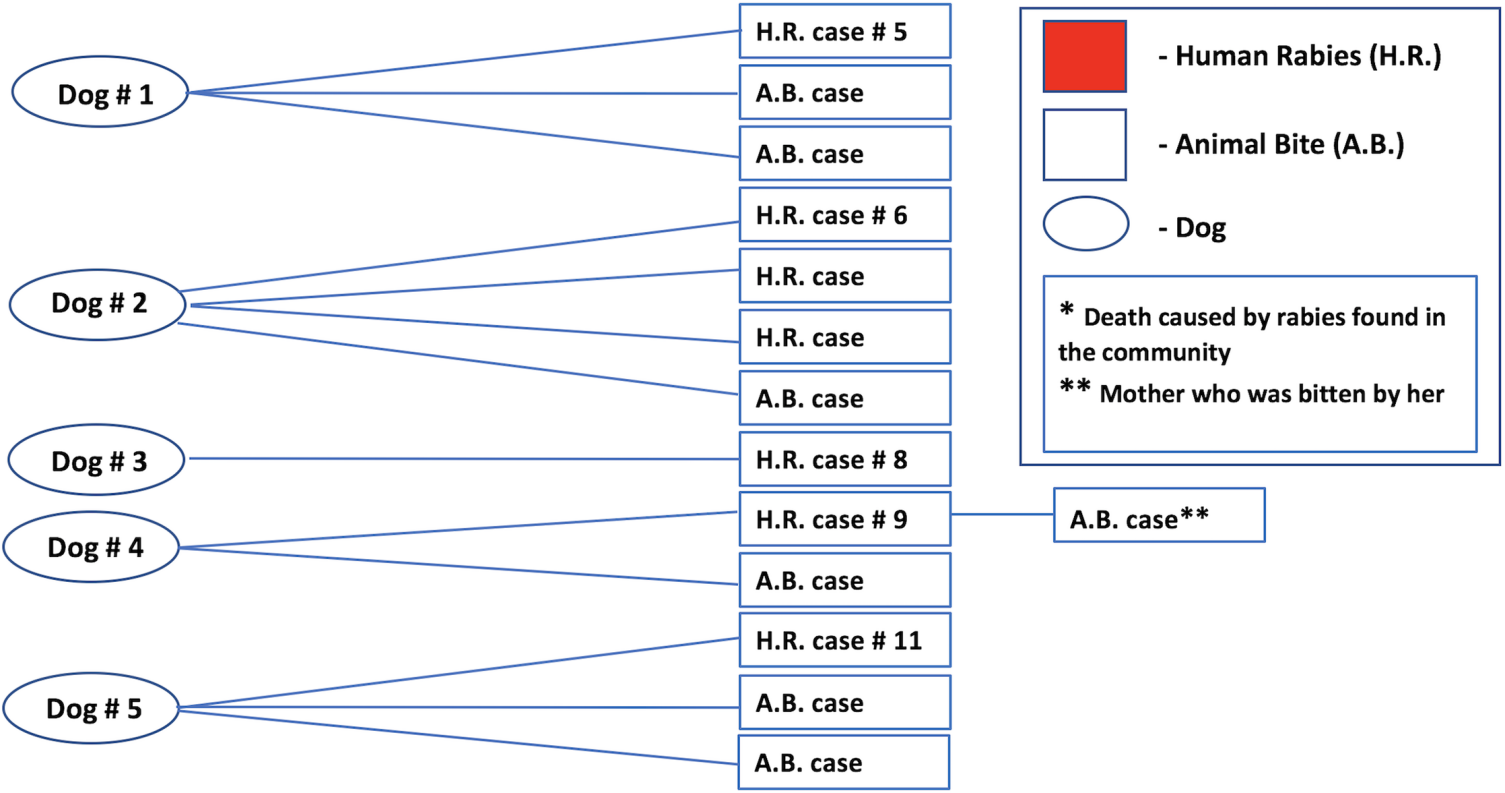
Epidemiological link between cases of human rabies admitted to Maputo Central Hospital and animal bite and human rabies cases identified at the community level. The left circles represent the dogs linked to cases initially identified at Maputo Central Hospital. The rectangles with numbered human rabies case (H.R. case #) inside at the right represent human rabies cases identified at Maputo Central Hospital. The rectangles at the right with non numbered human rabies case (H.R. case) or animal bite (A.B. case) represents cases of human rabies and animal bites identified during the community investigation.

During the community investigation, we were able to link seven cases of human rabies to bites of five dogs, as shown in Fig 4. Four of the five dogs were each linked to one rabies victim, and one was linked to three victims, two of whom were identified in the community investigation.

Fig 4 also shows that six other people were bitten by four of these rabid dogs, but did not develop rabies. Of these six cases, only one received post-exposure vaccination and none received RIG. Also, one rabid child had bitten his mother, but she did not develop rabies.

### Laboratory confirmation of rabies in suspected animals

Data collected from the Central Veterinary Laboratory in Maputo showed laboratory confirmed rabies in two dogs during the period under investigation.

### Risk factors associated with human rabies

Demographic variables and other known risk factors were compared between cases of animal bites and cases of human rabies in order to determine the predictive variables associated with a higher risk of developing rabies after an animal bite (Table 3). Age < 15 years (OR: 4.8, p = 0.05), a bite by a stray dog (OR: 13.6, p = 0.00), deep wound (OR: 6.1, p = 0.02), bite in the head (OR: 11.6; p = 0.00), bite by an unvaccinated dog (OR: 16.7, p = 0.01), failure to wash the bite site with soap and water (OR: 17.1, p = 0.00) and no post-exposure prophylaxis (OR: 16.7, p = 0.01), were all associated with the development of rabies.

**Table 3:** Case control study to assess risk factors for human rabies in Maputo city and province between April 1^st^ and August 14^th^ 2014.

Characteristics	Human Rabies n (%)	Non Rabies animal bite n (%)	OR	*P-value*
**Total**	14	58		
**Male victims**				
Yes	8 (57.1)	31 (53.5)	1.2	0.9
No	6 (42.9)	27 (46.5)		
Missing	0	0		
**Age year**				
Median	7.5	18.0		0.002
IQR	5.0–12.0	9.0–30.0		
**Children under 15 years**				
Yes	11 (78.6)	26 (44.8)	4.8	0.05
No	3 (21.4)	31 (53.5)		
Missing	0	1 (1.7)		
**Dog ownership status**				
Stray	7 (50.0)	9 (15.5)	13.6	0.00
Known	2 (14.3)	35 (60.4)		
Missing	5 (35.7)	14 (24.1)		
**Depth of the wound**				
Deep	8 (57.2)	17 (29.3)	6.1	0.02
Not deep	3 (21.4)	39 (67.2)		
Missing	3 (21.4)	2 (3.5)		
**Site of bite**				
Head	6 (42.9)	4 (6.9)	11.6	0.00
Not head	7 (50.0)	54 (93.1)		
Missing	1 (7.1)	0		
**Vaccination status of the dog**				
Not vaccinated	13 (92.9)	14 (24.2)	16.7	0.01
Vaccinated	1 (7.1)	18 (31.0)		
Missing	0	26 (44.8)		
**Cleaning of the wound after the bite**				
Not soap and water	5 (35.7)	7 (12.0)	17.1	0.00
Soap and water	2 (14.3)	48 (82.8)		
Missing	7 (50.0)	3 (5.2)		
**Post exposure vaccination**				
No	13 (92.9)	14 (24.2)	16.7	0.01
Yes	1 (7.1)	18 (31.0)		
Missing	0	26 (44.8)		

#### Comparison of cases of animal bites and human rabies notified through the routine surveillance system and collected through the investigation

Table 4 shows that in Maputo City, the number of cases of animal bites notified through the routine surveillance system between April and July 2014 was lower than the number identified during the outbreak investigation for the same period. Only data from Maputo City is presented, as data from Matola City was not discriminated from the rest of the province. Remarkably, none of the 12 cases of rabies identified from April to July were notified through the routine surveillance system.

**Table 4:** Comparison of cases of animal bites and human rabies notified through the routine surveillance system and detected during the investigation in Maputo city, April-July 2014.

Source of data	Animal bites	Human rabies
	Maputo City	Maputo City
Routine surveillance	279	0
During outbreak investigation	529	8

## Discussion

To our knowledge this represents the first detailed attempt to determine the epidemiological profile of human rabies and animal bites in Mozambique.

During the investigation of the outbreak in the capital city and adjoining provincial capital, none of the 14 cases of human rabies detected were notified through the routine surveillance system. These findings confirm our belief that the true number of cases of human rabies in Mozambique is much higher than reported. Under-reporting of rabies is common in low and middle income countries [5, 11].

In this outbreak, one reason for under-reporting was a failure to diagnose rabies. In other countries in sub-Saharan Africa, a lack of clinical awareness of rabies causes many cases to be misdiagnosed as non-rabies meningo-encephalitis [11–14]. A more regular training program directed to health professionals and incorporation of rabies into under graduate training curricula would avoid many misdiagnoses.

All cases were hospitalized, and the failure to notify was due to a breakdown in the surveillance system. In rural areas, the causes are more likely to be the same as in the rest of sub-Saharan Africa, where most deaths (>75%) occur in the community, and most victims seek traditional healers for medical care [15].

Our investigation showed that all cases of rabies were bitten by a dog and most animal bites were attributed to dogs. This was an expected finding and corroborates data from other countries [6, 16–18], suggesting that control efforts should be concentrated on dogs. Most of the dog bite victims and cases of human rabies resided in the suburban areas of Maputo and Matola cities. This could be explained by the higher population density, lower educational level and a higher proportion of stray and unvaccinated dogs [9]. Particular attention should be paid to the suburban areas of the cities, which represent a rapidly growing population in Mozambique. In other countries re-emergence of rabies has also been also associated with rapid urbanization [11, 19].

Animal bites and human rabies were slightly more frequent in males compared to females.

With regard to age distribution, 12 of the 14 cases of human rabies occurred in children under the age of fifteen years, as did 37.8% of the animal bites. This is in accordance with WHO estimates that most rabies cases and more than 40.0% of animal bites in Asia and Africa occur in children [1]. This increased risk may be attributed to children’s increased curiosity, with frequent provoking behavior and inexperience or lack of skills in dealing with aggressive dogs [16–18, 20, 21]. The high proportion of animal bites in this age group is also of concern because children are less likely to report minor dog bites or scratches to their caregivers and so are at higher risk of missing the opportunity to receive post-exposure prophylaxis [14, 22]. A study conducted in Nigeria showed that a higher level of education of school age children was correlated with a lower risk of rabies [22]. Education of children and their parents or guardians, particularly on the dangers of dog bites, could play a critical role in reducing the burden of rabies.

Almost one third of the animal bite victims and most of the human rabies cases in this study were bitten by a stray dog, corroborating findings from Nigeria and other developing countries, where the populations of stray dogs is rapidly increasing in urban areas [12, 14, 18]. Whether the number of stray dogs in Maputo City is increasing is not known, because no canine census has been conducted in recent years.

WHO recommends that coverage of canine immunization should be equal to, or greater than 70% [4, 23]. In our study, the vaccine coverage of biting dogs whose immunization status was known was low, at 53.8%. No recent study on canine immunization coverage is available, but a recent publication showed that Mozambique is ranked among the countries with the lowest expenditure on dog immunization in the region [8]. Factors that contribute significantly to the low level of canine immunization in developing countries including Mozambique are: i) lack of adequate public health laws on dog ownership and immunization, ii) poor implementation of existing legislation on dog ownership and immunization, iii) limited access to veterinary services for canine immunization and iv) irregular implementation of canine immunization campaigns, [22, 24, 25].

In our study no rabies victim of rabies had received complete post-exposure vaccination. Although records were poor, they showed that only a small proportion of animal bite victims had received post-exposure vaccination. This was mostly due to the absence of vaccine, although not all cases of dog bite were referred by health centers to the Prophylaxis Centers. Health workers also attempted to ration their small supply of vaccine, only giving two doses. A study in Tanzania showed that people may not return 5 times to complete the full course of prophylactic vaccine [26], but we do not know if this happened in these cases.

According to the national and international guidelines, the biting dog should be quarantined and monitored for the presence of any sign of canine rabies for up to 10 days and after that period, the health professionals should conduct a physical examination of the animal [1, 20, 27]. However, only a few dog owners returned to the Prophylaxis Center. This suggests that a more aggressive implementation of public health legislation is needed to ensure that dog owners return for the follow up visit.

We investigated the factors associated with the risk of developing rabies among those bitten and found that a lower age was associated with a significantly higher risk of developing rabies. Previous studies have shown that the small size of children contributes to their higher risk of developing rabies [28, 29]. A bite in the head and deep wounds were also associated with a higher risk of developing rabies, as previously shown [16, 21, 30, 31].

We also found that people who were bitten by an unknown or stray dog or by an unvaccinated dog were at higher risk to develop rabies, which reemphasizes that effective implementation of public health laws that reduce the number of stray dogs and force people to vaccinate their dogs represents a critical strategy to reduce the number of stray and unvaccinated dogs.

Poor management of wounds and poor adherence to post-exposure prophylaxis were also associated with a higher risk for developing human rabies after exposure. The health services need to improve their management of dog bites to prevent tragic deaths from rabies, such as those reported in this outbreak. Community education on preventive measures also has the potential to save many lives in Mozambique.

### Limitation

A major limitation of our study was the poor quality of records of cases of animal bites at the Prophylaxis Centers.

## Conclusions

In conclusion, our study shows that weak control measures, including low coverage rates of dog immunization, poor control of stray and unowned dogs, inadequate management of bites, and poor availability of and adherence to post-exposure prophylaxis, are major factors driving rabies outbreaks in Maputo and Matola cities. None of the cases of human rabies that occurred during the outbreak were notified through the routine surveillance system, showing that rabies is seriously under-reported. Overall, we recommend aggressive interventions that include regular canine mass vaccination campaigns, improvement of the provision of vaccines to humans and animals, accompanied by regular campaigns to remove stray dogs and massive education in communities and primary schools.

## Supporting information

S1 Appendix Case investigation form. (PDF)
