# Supplementary material for: Epidemiology, clinical features and risk factors for human rabies and animal bites during an outbreak of rabies in Maputo and Matola cities, Mozambique, 2014: Implications for public health interventions for rabies control
Source: PLoS Negl Trop Dis. 2017 Jul 24;11(7):e0005787. doi: 10.1371/journal.pntd.0005787 (PMC5542695; doi:10.1371/journal.pntd.0005787)
Supplement: S1 Appendix — (PDF) [file pntd.0005787.s001.pdf]

FICHA DE INVESTIGAÇÃO DE CASO DE MORDEDURA POR ANIMAL

CASO Nº_____

Data da notificação___/___/___ Data da investigação___/___/___ Data da agressão___/___/___

1. IDENTIFICAÇÃO DA VÍTIMA

Nome da vítima:______________________ Sexo____ Idade______ anos
Residência no Bairro:______________________ Quarteirão_______ Casa número______
Av./Rua:______________________ Localidade______________________ Perto de ______________
Telefone________________ Celular________________ Centro de Saúde________________
Área de Saúde________________ Distrito________________ Província________________

2. DADOS SOBRE A LESÃO

Tipo de agressão
- Arranhão ou rasgadura
- Lambidela na pele sã ☐
- Lambidela na pele com ferida ☐
- Lambidela da mucosa com ferida ☐
- Mordedura única ☐
- Mordeduras múltiplas ☐
- Sem contacto ☐
- ☐

Localização da lesão
- Cabeça ☐
- Face ☐
- Pescoço ☐
- Tórax ☐
- Abdómen ☐
- Braço ☐
- Antebraço ☐
- Mão ☐
- Nádega ☐
- Coxa ☐
- Perna ☐
- Pé ☐

Tipo de lesão
1. Profunda ☐
2. Superficial ☐
3. Sem lesão ☐

O animal mordeu
1. Legítima defesa ☐
2. Expontâneo ☐
3. Com provocação ☐
4. Sem provocação ☐

3. TRATAMENTO DA (S) FERIDA(S)

Água e sabão ☐
Desinfectante ☐
Sutura ☐

Avaliação da(s) lesão(ões)

Leve ☐
Grave ☐

4. DADOS SOBRE O ANIMAL

Doméstico ☐ Vádio ☐ S/inf ☐

Se é vadio

Em que bairro se deu a agressão______________________
Rua/Av.______________________

Espécie de animal

Cão ☐ Gato ☐ Macaco ☐ Morcego ☐
Cão polícia ☐ Burro ☐ Outro ☐

Se é Doméstico

Nome do dono______________________
Bairro______________ R/Av.______________
Quarteirão_____ Nº da casa_______ Tel:_________

Estado vacinal do animal

Data da última vacina___/___/___ tipo de vacina_____
______________ Lote nº______ Validade___/___/___
Distrito______________ Província______________

Observação do Animal

Impossível fazer ☐ Ao domicílio ☐
Foi possível observar ☐ Num canil ☐

Situação da animal após 10 dias

São ☐ Raivoso ☐
Morto ☐ Não sabe ☐

É necessário vacinar a pessoa agredida? Sim ☐ Não ☐

Tipo de vacina______________________ IgAR/____/___/___ VAT___/___/___/ Antibiótico___

OBSERVAÇÕES______________________

O INVESTIGADOR:______________________
CARGO:______________________ **Data:**___/___/___
